# The effect of gadolinium-based contrast-agents on automated brain atrophy measurements by FreeSurfer in patients with multiple sclerosis

**DOI:** 10.1007/s00330-021-08405-8

**Published:** 2022-01-03

**Authors:** Ingrid Anne Lie, Emma Kerklingh, Kristin Wesnes, David R. van Nederpelt, Iman Brouwer, Øivind Torkildsen, Kjell-Morten Myhr, Frederik Barkhof, Lars Bø, Hugo Vrenken

**Affiliations:** 1 Department of Clinical Medicine, University of Bergen, Bergen, Norway; 2 Department of Radiology and Nuclear Medicine, MS Center Amsterdam, Amsterdam Neuroscience, Amsterdam UMC, Location VUmc, De Boelelaan 1118, 1081 HZ Amsterdam, The Netherlands; 3 Neuro-SysMed, Department of Neurology, Haukeland University Hospital, Bergen, Norway; 4 Department of Neurology, St. Olav’s University Hospital, Trondheim, Norway; 5 Institutes of Neurology and Healthcare Engineering, UCL London, London, UK; 6 Norwegian Multiple Sclerosis Competence Centre, Department of Neurology, Haukeland University Hospital, Bergen, Norway

**Keywords:** Multiple sclerosis, Magnetic resonance imaging, Gadolinium, Atrophy, Grey matter

## Abstract

**Objective:**

To determine whether reliable brain atrophy measures can be obtained from post-contrast 3D T1-weighted images in patients with multiple sclerosis (MS) using FreeSurfer.

**Methods:**

Twenty-two patients with MS were included, in which 3D T1-weighted MR images were obtained during the same scanner visit, with the same acquisition protocol, before and after administration of gadolinium-based contrast agents (GBCAs). Two FreeSurfer versions (v.6.0.1 and v.7.1.1.) were applied to calculate grey matter (GM) and white matter (WM) volumes and global and regional cortical thickness. The consistency between measures obtained in pre- and post-contrast images was assessed by intra-class correlation coefficient (ICC), the difference was investigated by paired t-tests, and the mean percentage increase or decrease was calculated for total WM and GM matter volume, total deep GM and thalamus volume, and mean cortical thickness.

**Results:**

Good to excellent reliability was found between all investigated measures, with ICC ranging from 0.926 to 0.996, all *p* values < 0.001. GM volumes and cortical thickness measurements were significantly higher in post-contrast images by 3.1 to 17.4%, while total WM volume decreased significantly by 1.7% (all *p* values < 0.001).

**Conclusion:**

The consistency between values obtained from pre- and post-contrast images was excellent, suggesting it may be possible to extract reliable brain atrophy measurements from T1-weighted images acquired after administration of GBCAs, using FreeSurfer. However, absolute values were systematically different between pre- and post-contrast images, meaning that such images should not be compared directly. Potential systematic effects, possibly dependent on GBCA dose or the delay time after contrast injection, should be investigated.

**Trial registration:**

Clinical trials.gov. identifier: NCT00360906.

**Key Points:**

*• The influence of gadolinium-based contrast agents (GBCAs) on atrophy measurements is still largely unknown and challenges the use of a considerable source of historical and prospective real-world data.*

*• In 22 patients with multiple sclerosis, the consistency between brain atrophy measurements obtained from pre- and post-contrast images was excellent, suggesting it may be possible to extract reliable atrophy measurements in T1-weighted images acquired after administration of GBCAs, using FreeSurfer.*

*• Absolute values were systematically different between pre- and post-contrast images, meaning that such images should not be compared directly, and measurements extracted from certain regions (e.g., the temporal pole) should be interpreted with caution.*

**Supplementary Information:**

The online version contains supplementary material available at 10.1007/s00330-021-08405-8.

## Introduction

Grey matter (GM) atrophy measured on MRI in persons with multiple sclerosis (MS) reflects irreversible neuroaxonal loss and neurodegenerative changes in the CNS [1]. The degree of GM atrophy has been shown to consistently correlate with physical [2, 3] and cognitive [4] disability, and is regarded as a promising neurodegenerative biomarker. Furthermore, as the demand for neuroprotective interventions increases, GM atrophy is an easily available outcome measure [5–7].

There are a number of available methods and software to measure GM atrophy. Although FreeSurfer requires substantial processing time, making it less suitable for clinical practice, it is one of the most commonly used automated methods in research, especially for cortical parcellation and thickness estimation. FreeSurfer is publicly available and widely validated [8–12], and in the body of literature on GM atrophy in MS, many key papers have used FreeSurfer [13–15].

Due to the high tissue contrast [16–18] in unenhanced three-dimensional (3D) T1-weighted images, this image type is commonly used by brain segmentation software and required by FreeSurfer, as well as other software with similar purposes. However, unenhanced 3D T1-weighted images are not mandatory in suggested standardised brain MRI protocols for MS [19] and may not be routinely included. Instead, post-contrast T1-weighted images are often prioritised, especially in clinical settings. In case of ongoing inflammation, the intravenously administered contrast agent leaks into the brain parenchyma in locations where the blood–brain barrier (BBB) is disrupted [20]. These post-contrast images are valuable both in baseline and follow-up examinations, as they can unequivocally detect lesions with active inflammation [19].

The contrast agent used is almost universally gadolinium-based, consisting of a central paramagnetic Gd^3+^ ion chelated to a carrier molecule to prevent the toxicity of free Gd^3+^, while still maintaining its paramagnetic properties. Gadolinium-based contrast agents (GBCAs) shorten both the longitudinal (T1) and transverse (T2) relaxation times [21], leaving areas in which GBCAs accumulate as bright or hyperintense compared to surrounding tissue on T1-weighted images.

The use of GBCAs has increased over the last three decades [22], making up a considerable source of historical and prospective real-world data. However, the value of such data for brain atrophy measurements depends on our ability to correctly interpret the data in automated image analyses. The influence of GBCAs on atrophy measurements is still largely unknown and has previously been investigated in only a few studies using different image analysis techniques [23, 24]. In this study, our aim is to validate the use of post-contrast T1-weighted images for volume and cortical thickness measurements and to provide guidelines on how to interpret results from clinically relevant and commonly considered measures. To do so, total WM and GM volume, total deep GM and thalamus volume, and mean cortical thickness measures were obtained in pre- and post-contrast images by FreeSurfer and compared.

## Materials and methods

### Participants

The patients included in this study participated in a 10-year follow-up visit following a multi-centre, randomised, placebo-controlled trial of ω-3 fatty acids in MS (the OFAMS-study), which has previously been described in detail [25]. A total of 85 of the 92 persons with relapsing–remitting MS (RRMS) [26] originally enrolled in the OFAMS-study participated in the 10-year follow-up visit and underwent clinical, biochemical, and radiological examinations at their local study site.

The study was approved by the Regional Committee for Medical and Health Research Ethics in Western Norway Regional Health Authority (clinical trials.gov, identifier: NCT00360906). All participants gave their written informed consent.

### MRI data and analysis

#### MRI data acquisition

Imaging at the 10-year follow-up visit was performed at the different study sites, on a 3-Tesla (T) MRI scanner if available, alternatively using a 1.5 T MRI scanner, with a standard head coil. The acquisition included a post-contrast sagittal 3D T1-weighted sequence; acquisition details across sites are provided in Table 1. Furthermore, a sagittal T2-weighted 3D fluid-attenuated inversion recovery (FLAIR) sequence was acquired according to locally optimised protocols. The full MRI protocol provided to the study sites is available in eAppendix 1. The study sites were encouraged to include the same 3D T1-weighted sequence before contrast-agent administration, if possible. For the present study, only the subset of the participants who underwent 3D T1-weighted MR imaging both before and after injection of GBCAs, during the same scanner visit, and with the exact same acquisition protocol, was included.

**Table 1:** Details on MRI acquisition per protocol

Protocol (number of patients)	1 (3)	2 (3)	3 (3)	4 (3)	5 (5)	6 (2)	**7** (3)
Scanner	Siemens Aera	Siemens Skyra	Siemens Avanto	Siemens Aera	Philips Achieva	Siemens Prisma	Philips Achieva
Field strength	1.5 T	3 T	1.5 T	1.5 T	1.5 T	3 T	1.5 T
3DT1 sequences	MPRAGE	MPRAGE	MPRAGE	MPRAGE	FFE	MPRAGE	FFE
TR (ms)	1940	2300	2200	2200	7.6	1800	7.1
TE (ms)	2.69	2.32	2.82	2.67	3.75	2.28	2.2
TI (ms)	976	900	900	900		900	
Flip angle (°)	8	8	8	8	8	8	8
Voxel size	1.00 × 0.98 × 0.98	0.9 × 0.94 × 0.94	1.00 × 0.98 × 0.98	1.00 × 0.98 × 0.98	1.00 × 0.98 × 0.98	1.00 × 0.50 × 0.50	1.00 × 1.00 × 1.00
Head receiver coil	Unknown	Unknown	HE1-4	HE1-4	SENSE-head-8	Unknown	SENSE-head-8
GBCA	Gadoterate meglumine	Gadoterate meglumine	Gadoterate meglumine	Gadoteridol	Unknown	Gadoterate meglumine	Unknown

Abbreviations: *TR*, repetition time; *TE*, echo time; *TI*, inversion time; *ms*, millisecond; *mm*, millimetre; *MPRAGE*, magnetization-prepared rapid gradient-echo; *FFE*, fast field echo; *GBCA*, gadolinium-based contrast agent

#### MRI data processing

##### Lesion segmentation and lesion filling

Lesion segmentation was done on FLAIR images using lesion segmentation tool (LST) (version 2.0.15; http://applied-statistics.de/lst.html) [27]. The lesion probability map in FLAIR space was brought to T1-weighted space by FLIRT linear registration of the FLAIR image to the T1 image, using 7 degrees of freedom, correlation ratio as the cost function, and trilinear interpolation. Afterwards, a threshold of 0.1 was used to binarise the lesion probability map. To optimise the lesion filling, gadolinium-enhancing regions (both lesions and other regions) were first removed, by applying an upper-intensity threshold at the 98^th^ percentile. Next, the FMRIB Software Library (FSL) (version 5.0.10; http://www.fmrib.ox.ac.uk/fsl) was used to fill in lesional voxels in the T1-weighted images using the lesion_filling tool [28], and these filled lesions were pasted into the original post-contrast 3D T1-weighted images.

##### Morphological reconstruction

Cortical reconstruction and parcellation for cortical volume and thickness measurement and subcortical segmentation were performed with FreeSurfer, a freely available software package for academic use, available through online download (http://surfer.nmr.mgh.harvard.edu/). The findings presented here were obtained using FreeSurfer version 7.1.1; highly comparable findings obtained using FreeSurfer version 6.0.1 are presented in Table e1. The technical details of FreeSurfer procedures have been previously described [29, 30] and briefly summarised in eAppendix 2.

Quality control was performed by visual inspection, and any segmentation errors were recorded for each patient. In cases where only specific anatomical regions were incorrectly segmented, we chose to not apply any corrections for these errors in our analyses.

The Desikan-Killiany atlas [31] was used to extract cortical thickness measures (mean cortical thickness, left and right hemisphere) and to study regional differences in cortical thickness between pre- and post-contrast images, across subjects, by creating a heat map. Furthermore, total cerebral GM and WM volume and total deep GM and thalamus volume (left and right hemisphere) were obtained.

##### MRI quality control tool

To investigate potential root causes of any observed segmentation differences, both pre- and post-contrast T1-weighted images were analysed using the MRI Quality Control Tool (MRIQC) [32]. MRIQC is an open-source software and extracts no-reference image quality metrics (IQMs) from structural and functional MRI data [32]. Using a segmentation into GM, WM, and CSF by FSL-FAST [33], MRIQC calculates tissue-specific signal-to-noise ratio (SNR) values as well as the contrast-to-noise ratio (CNR) between GM and WM. Additionally, based on these values obtained from MRIQC, the contrast ratio (CR) between white and grey matter was also calculated.

### Statistical analysis

Statistical analyses were performed using the Statistical Product and Service Solutions (SPSS) for macOS (Version 25; SPSS). Data were visually and statistically examined using the Kolmogorov–Smirnov test for normality. To assess the agreement between volume and thickness measurements obtained before and after GBCA administration, the intra-class correlation coefficient (ICC) was determined, based on a mean rating (*k* = 2), consistency, two-way mixed model. Scatterplots were created to visualise the agreement. To assess whether any systematic differences in structural measurements or IQMs were present between pre- and post-contrast measurements, paired t-tests were performed. Furthermore, boxplots were made to illustrate any differences, and Bland–Altman plots were created to identify fixed or proportional bias [34]. As an exploratory analysis, paired t-tests were used to investigate a possible systematic difference between field strengths (1.5 and 3 T).

## Results

Pre- and post-contrast T1-weighted images were obtained with the exact same acquisition protocol in a total of 23 patients. One patient was excluded due to a large image artifact, causing segmentation errors. Table 2 provides an overview of the demographic and clinical characteristics of the patient group.

**Table 2:** Demographic and clinical characteristics

Age in years, mean (SD)	50.5 (7.83)
Sex, female, *N* (%)	15 (68.2%)
Disease duration, mean in years (SD) / median (range)	13.8 (3) / 13 (12–25)
EDSS, mean (SD) / median (range)	2.9 (1.2) / 2.5 (1–6)
Disease phenotype (N)	RRMS (21), SPMS (1)
Study site (number of patients)	Site 1 (3), Site 2 (3), Site 3 (3), Site 4 (8 (2 scanners)), Site 5 (2), Site 6 (3)

Abbreviations: *SD*, standard deviation; *EDSS*, Expanded Disability Status Scale; *RRMS*, relapsing-remitting multiple sclerosis; *SPMS*, secondary progressive multiple sclerosis

### Quality control of FreeSurfer segmentations

All 22 pairs of pre- and post-contrast T1-weighted images finished the fully automated FreeSurfer pipeline (i.e., no hard failures). The most common soft failures (i.e., failures that do not disrupt the pipeline, but may need modification) are summarised in Table 3.

**Table 3:** Summary of the most common soft failures

Description	Frequency
The pial surface (representing the border between cortical GM and CSF) or the border of segmented deep GM structures, expanding into extraparenchymal tissue, including components of dura or blood vessels as part of the cortex or deep GM structures (Figs. 1b and 2)	Found in all scans, both pre- and post-contrast, but more frequently and to a more severe degree in post-contrast images
The pial surface failing to follow the white surface, causing “looping” errors (Fig. 1a) and subsequent incorrect enlargement of the cortical volume and thickness	Found in all scans, both pre- and post-contrast, but more frequently and to a more severe degree in post-contrast images
The constructed surface border between WM and GM (the white surface) failing to follow the intensity gradient correctly in the temporal poles, resulting in a suboptimal segmentation (Fig. 3)	Found to a moderate degree in two post-contrast images, and to a minor degree in a total of eight patients, in the post-contrast image in all eight, and in the pre-contrast image in three of those eight

Abbreviations: *GM*, grey matter; *CSF*, cerebrospinal fluid; *WM*, white matter

**Fig. 1:**
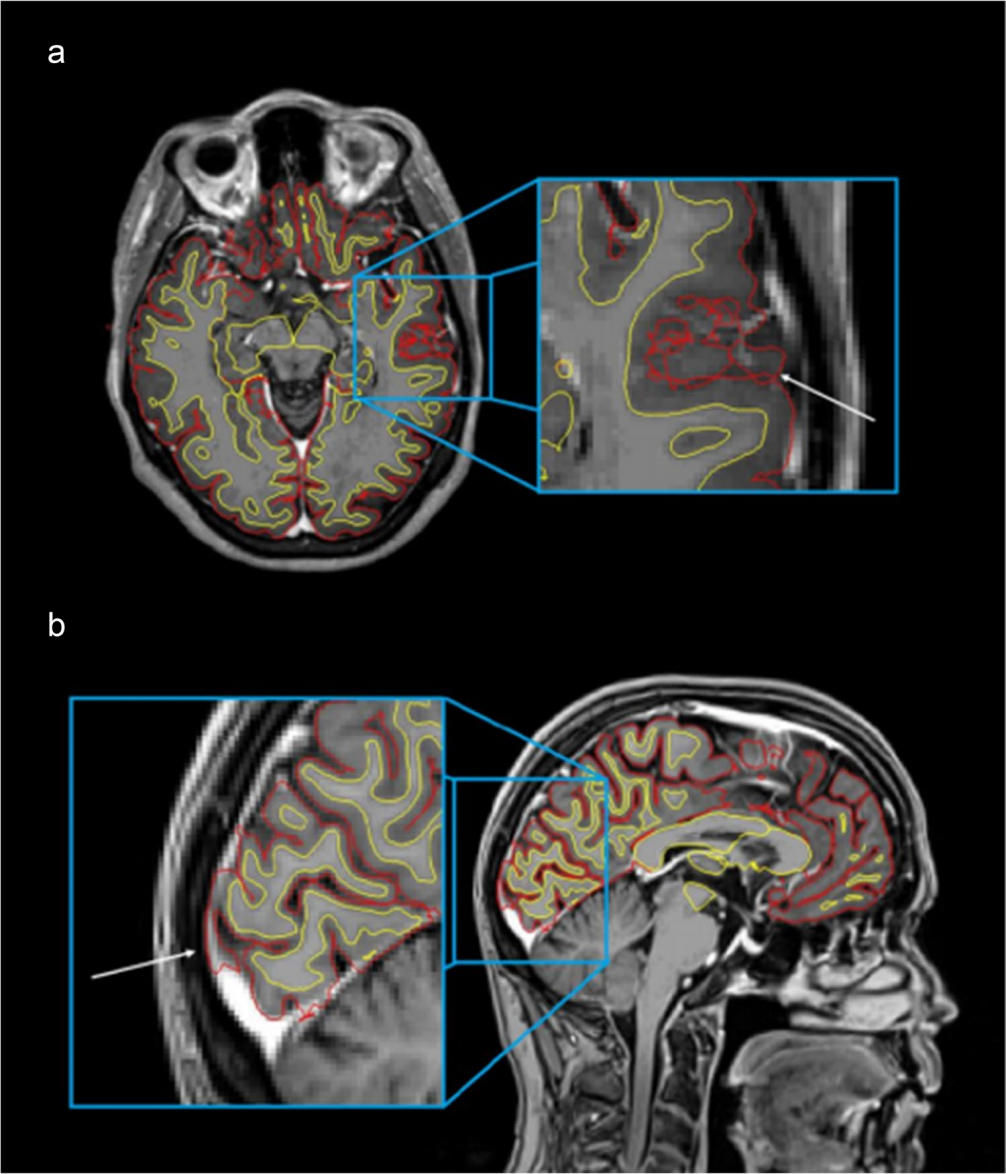
Post-contrast T1-weighted MRI, showing the border between WM and GM (white surface) (yellow), and the border between GM and CSF (pial surface) (red). **a** Axial slice showing a moderate pial surface “looping error” (white arrow). **b** Sagittal slice showing a typical skull stripping failure; a moderate error of the pial surface expanding into the dura and the sagittal sinus (white arrow)

**Fig. 2:**
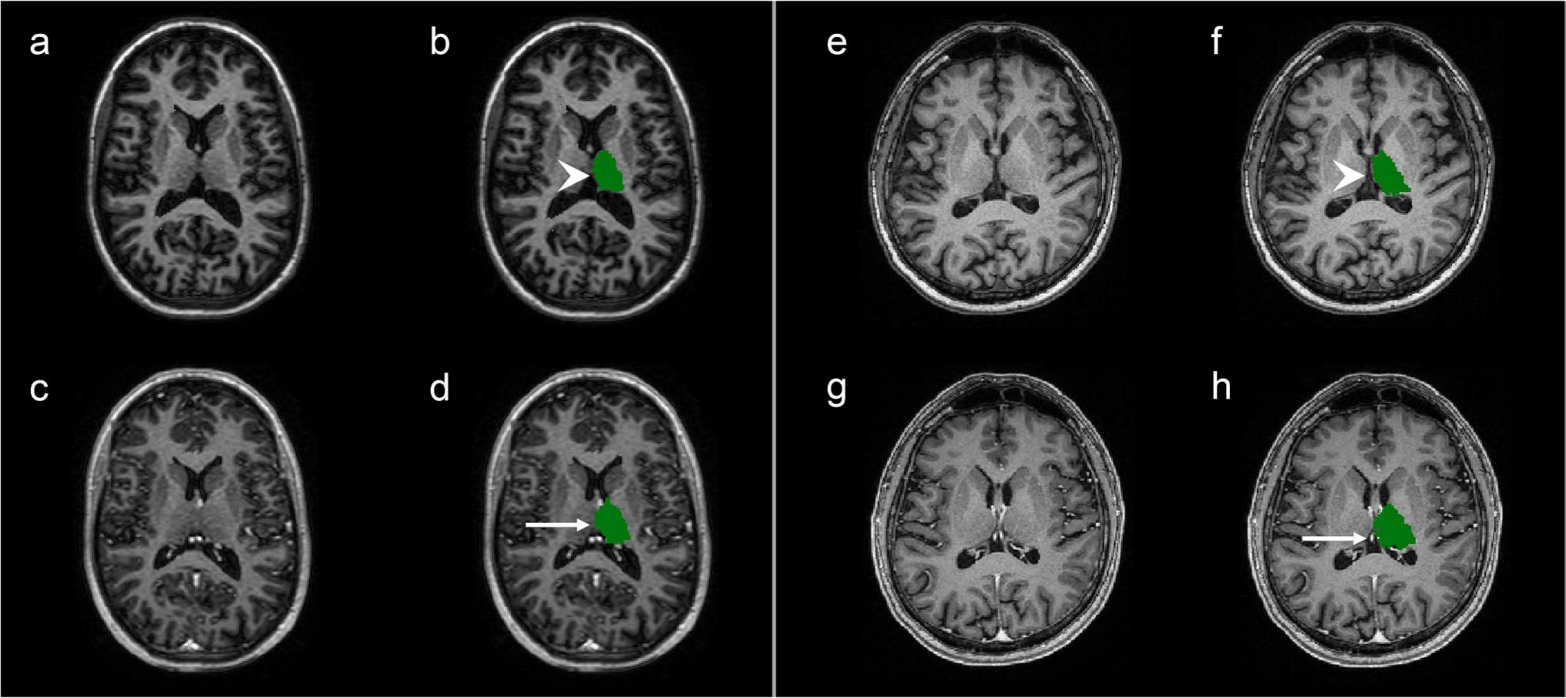
T1-weighted MRI, showing the segmentation of the left Thalamus in pre- and post-contrast images, in two different patients (subject E3 (**a**–**d**) and subject C1 (**e**–**h**)). **a–d** Axial slices demonstrating the typical quality of thalamus segmentations. In post-contrast images (**c**–**d**), the medial border of the left Thalamus is slightly overestimated (arrow) compared to pre-contrast images (arrowhead) (**a**–**b**), most likely due to hyperintense signal from extraparenchymal structures in the midline. **e**–**h** Axial slices demonstrating a more severe overestimation of the medial border of the left Thalamus (arrow) in post-contrast images (**g**–**h**) compared to pre-contrast images (arrow head) (**e**–**f**). Again, the segmentation of the medial border is overestimated due to inclusion of extraparenchymal hyperintense structures, in this case, the internal cerebral vein)

**Fig. 3:**
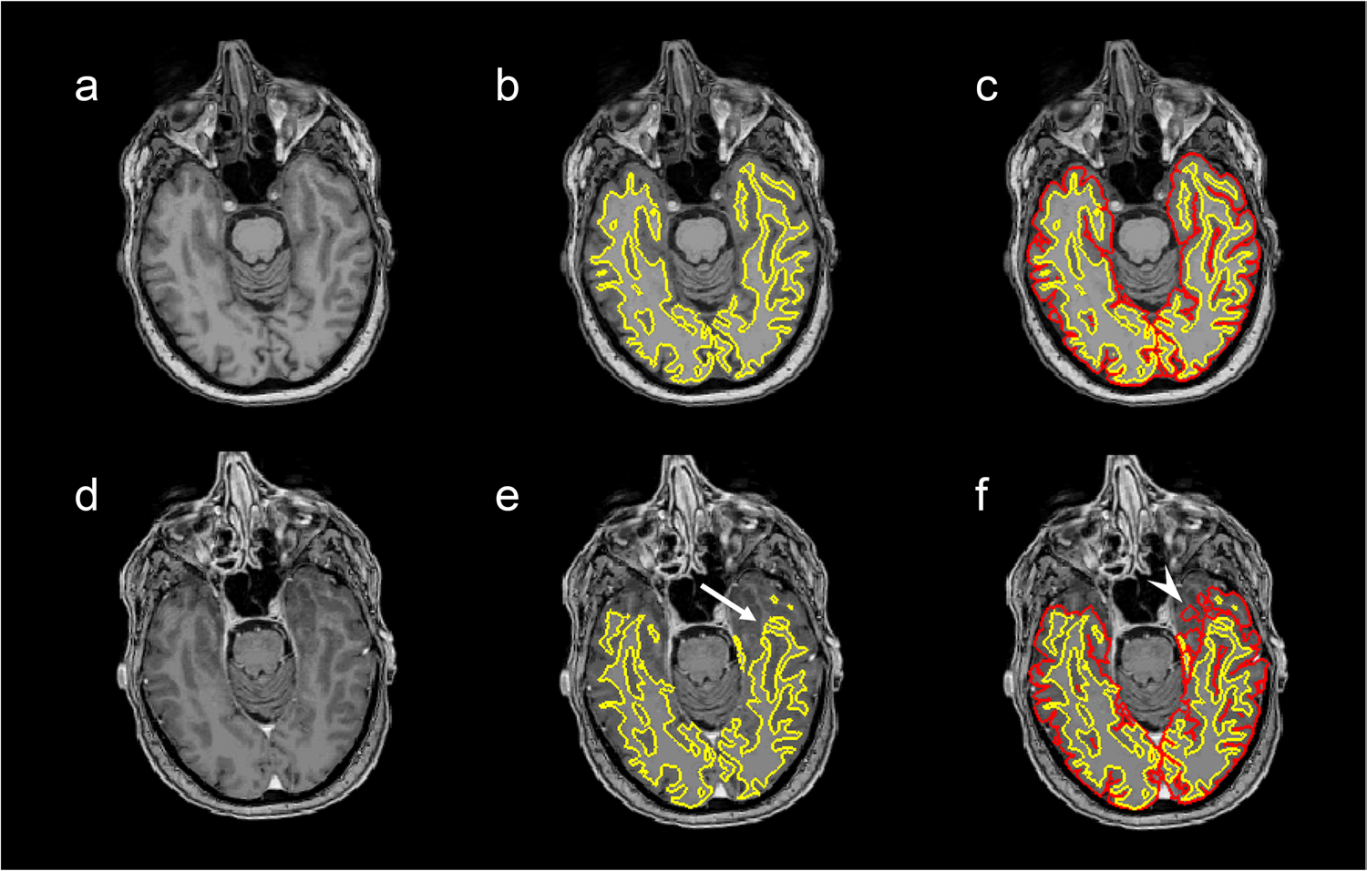
Pre-contrast (**a**–**c**) and post-contrast (**d**–**f**) T1-weighted images obtained from the same patient (subject A3) in the same MRI session. **b** and **e** show the white surface, which is the border between white and grey matter as automatically constructed by FreeSurfer (yellow). **c** and **f** show the pial surface, which is similarly the automatically constructed border between grey matter and cerebrospinal fluid (red), derived from the white surface. The figure demonstrates a typical failure of moderate degree, where the white surface fails to include parts of the temporal poles in the post-contrast image (**e**) (arrow), with subsequent mistakes in the pial surface (**f**) (arrowhead)

### Volume and cortical thickness measurements before and after administration of GBCAs

**Fig. 4:**
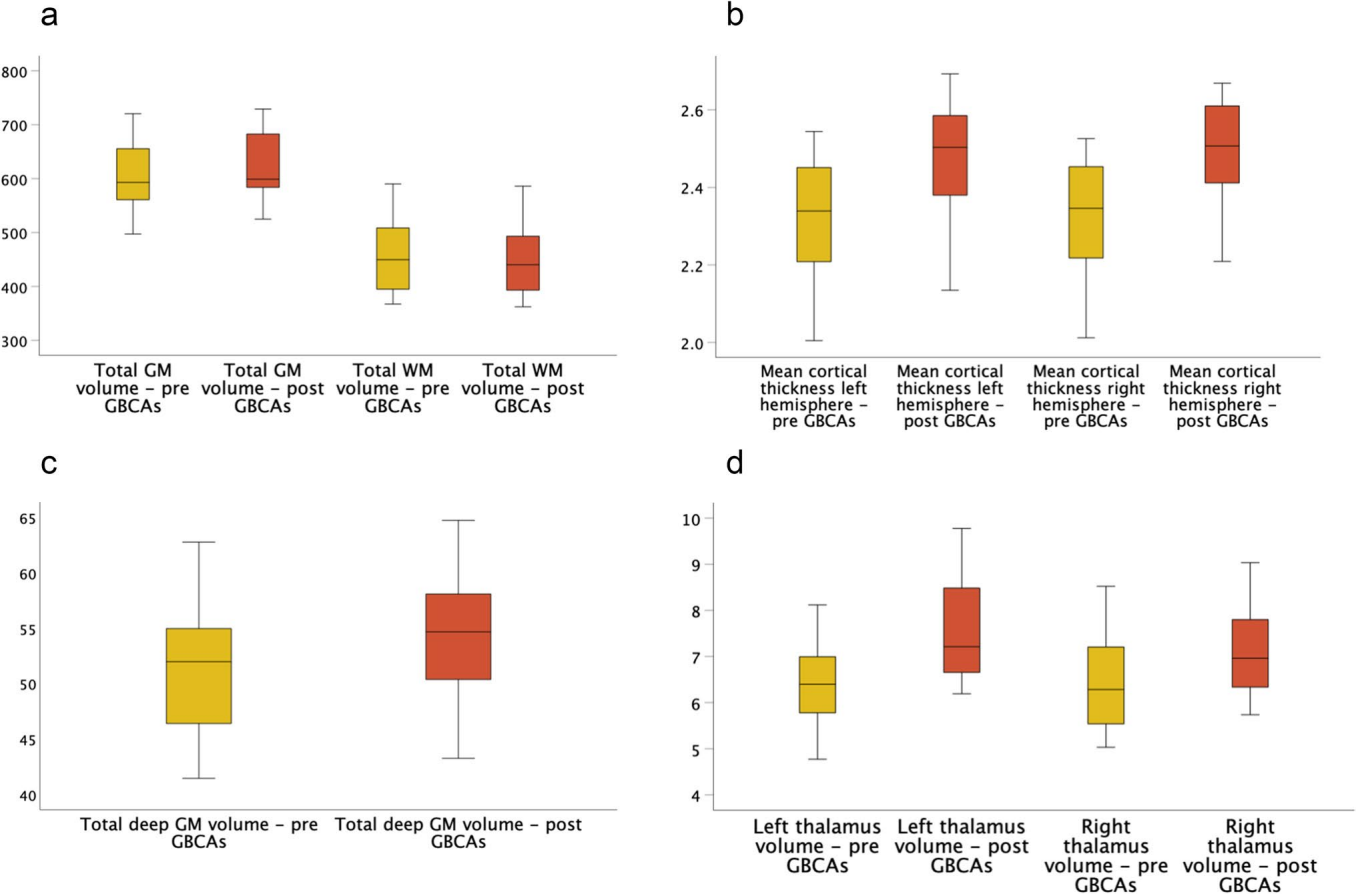
Boxplots of MRI measurements obtained before (yellow) and after (red) GBCA administration, in mL (**a**, **c**, **d**) and mm (**b**)

The mean values of MRI measurements obtained before and after GBCAs are summarised in Table 4 and Fig. 4. Briefly, a mean increase in GM volumes and cortical thickness measures were observed in post-contrast images, while a mean decrease was observed in total WM volume. The results of the exploratory analysis subdivided according to field strength are presented in Table e2, showing no clear systematic differences between field strengths.

**Table 4:** MRI measurement values

MRI measure	Mean value pre-contrast (SD)	Mean value post-contrast (SD)	Mean difference^a^ (SD)	Percent increase/decrease (SD)	ICC (95% confidence interval)
Total grey matter volume (mL)	602.53 (62.42)	620.33 (59.97)	17.80 (16.20)**	+ 3.06 (2.79) %	0.982 (0.957–0.993)
Total white matter volume (mL)	457.06 (63.05)	448.70 (59.25)	− 8.36 (7.35)**	− 1.74 (1.48) %	0.996 (0.991, 0.998)
Total deep grey matter volume (mL)	51.57 (5.90)	54.73 (5.61)	3.16 (2.04)**	+ 6.33 (4.43) %	0.968 (0.922–0.987)
Left thalamus volume (mL)	6.47 (0.93)	7.58 (1.09)	1.10 (0.48)**	+ 17.39 (8.46) %	0.940 (0.855–0.975)
Right thalamus volume (mL)	6.37 (0.99)	7.12 (0.92)	0.75 (0.50)**	+ 12.52 (9.04) %	0.926 (0.823–0.969)
Mean cortical thickness left hemisphere (mm)	2.32 (0.16)	2.49 (0.15)	0.17 (0.06)**	+ 7.38 (2.78) %	0.964 (0.913, 0.985)
Mean cortical thickness right hemisphere (mm)	2.33 (0.14)	2.49 (0.14)	0.16 (0.05)**	+ 7.13 (2.61) %	0.961 (0.906, 0.984)

Abbreviations: *SD*, standard deviation; *ICC*, intra-class correlation coefficient for consistency; *mL*, millilitres; *mm*, millimetre

^a^ Paired t-test

^**^p < 0.001

### Consistency of measurements obtained before and after administration of GBCAs

**Fig. 5:**
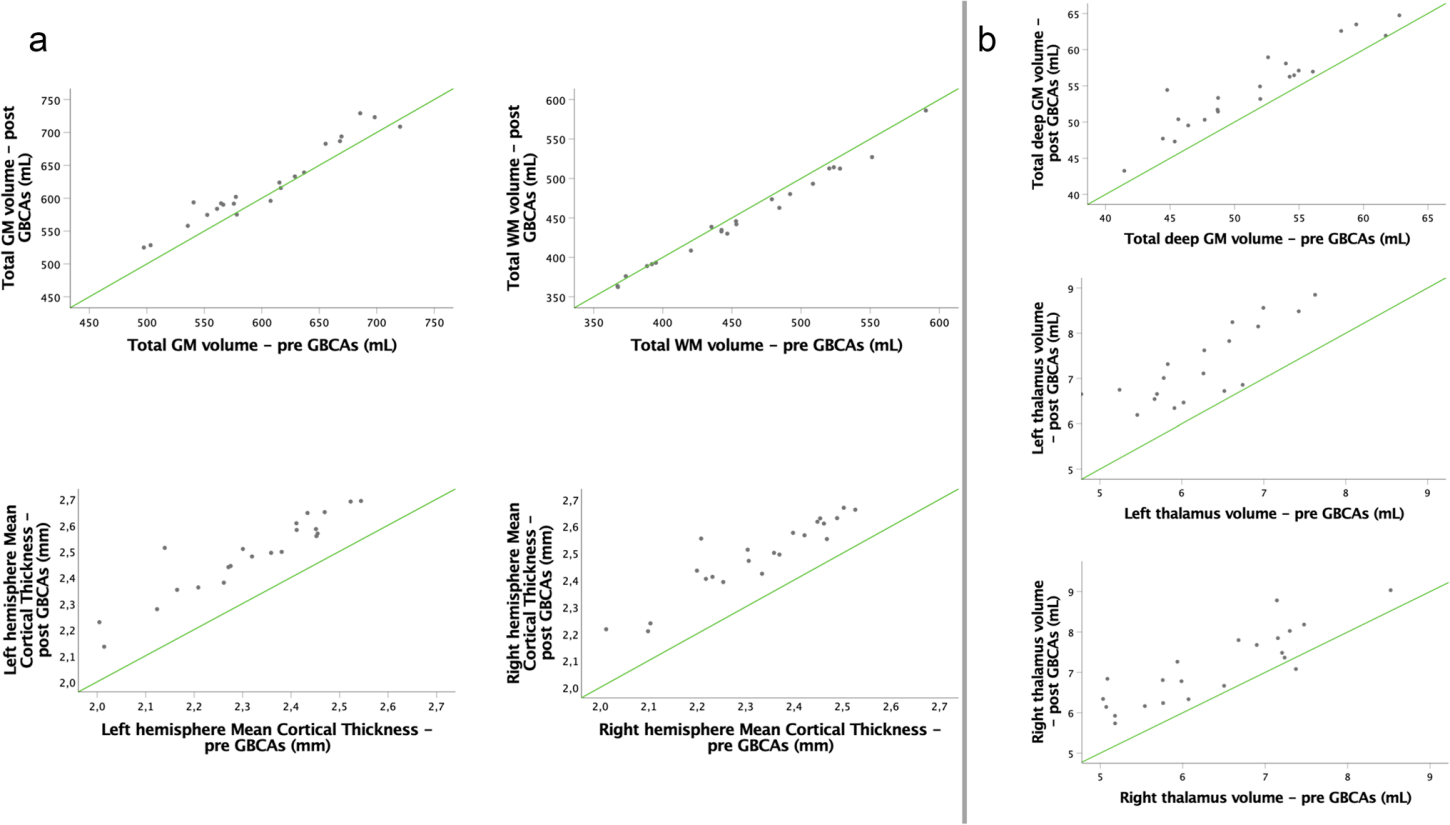
Scatterplots of global (**a**) and regional (**b**) MRI measurements obtained before and after GBCA administration. The green lines indicate identity lines

A high degree of reliability was found between the measurements obtained pre- and post-contrast, for all volumes and cortical thickness measures assessed. All ICC values (Table 4) were above 0.92, with the lowest values in the thalami, and above 0.96 for all larger structures, all *p* values < 0.001. The consistency between the measurements is demonstrated in Fig. 5.

### Difference in measurements before and after administration of GBCAs

GM volumes and mean cortical thickness were significantly higher after administration of GBCAs, in all investigated structures (Table 4, Figs. 4 and 5).

**Fig. 6:**
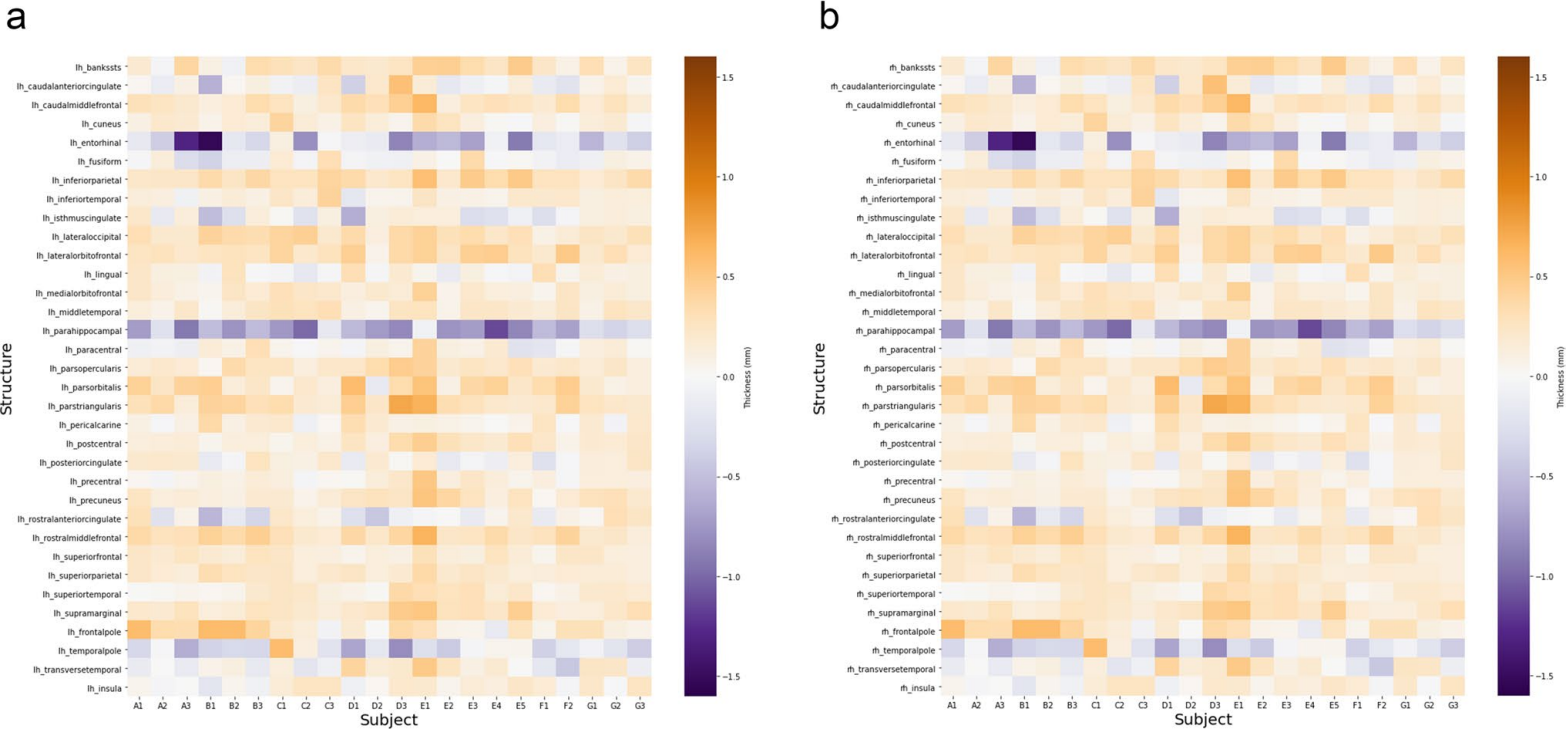
Heatmaps demonstrating the difference (mm) in cortical thickness in the left (**a**) and right (**b**) hemisphere after administration of GBCAs. Brown colours indicate an increase in cortical thickness, and purple colours indicate a decrease in cortical thickness (colour range between -1.6 mm and + 1.6 mm cortical thickness difference). Letters in subject names indicate MRI scanner (**a**–**g**)

Figure 6 shows heatmaps visualising the difference in cortical thickness between pre- and post-contrast images, demonstrating the general increase in thickness measured in post-contrast T1-weighted images. However, in a few exceptions, most prominently the temporal pole, the parahippocampal, and the entorhinal gyrus in the temporal lobe, cortical thickness decreased.

While GM volumes and cortical thickness measurements were higher after administration of GBCAs, total WM volume was significantly lower. Figure e1 in the supplementary material shows the constructed Bland–Altman plots, revealing systematic differences, but no proportional bias.

IQMs are reported in Table 5. The CNR was not significantly different between pre- and post-contrast images. Tissue-specific SNRs were significantly lower in post-contrast images, for both GM (*p* < 0.01) and WM (*p* < 0.0001). The CR between WM and GM was significantly higher in post-contrast images (*p* < 0.006).

**Table 5:** Image quality metrics obtained by MRI Quality Control Tool

Image quality metric	Mean value pre-contrast (SD)	Mean value post-contrast (SD)	Mean difference^a^ (SD)
CNR	3.23 (0.56)	3.25 (0.40)	− 0.1 (0.52)
SNR GM	10.89 (2.81)	9.02 (2.50)	1.87 (2.14)*
SNR WM	18.01 (3.72)	16.24 (3.56)	1.77 (1.64)***
CR	0.39 (0.08)	0.45 (0.08)	− 0.05 (0.08)*

Abbreviations: *SD*, standard deviation; *CNR*, contrast to noise ratio; *SNR*, signal to noise ratio; *GM*, grey matter; *WM*, white matter; *CR*, contrast ratio

^a^ Paired t-test

^*^p < 0.01, ***p < 0.0001

## Discussion

Our results demonstrate that using FreeSurfer, reliable GM volume- and cortical thickness measurements may be obtained from post-contrast 3D T1-weighted images. Despite systematic overestimation of the GM, high consistency was observed between all investigated MRI brain measurements obtained before and after administration of GBCAs.

To our knowledge, this is one of the very few studies investigating the effect of GBCAs on volume measures in MS patients and the first using FreeSurfer. In our study, when investigating the consistency between the measures obtained before and after administration of GBCAs, a good to excellent [35] reliability was found between all investigated measures. This is in agreement with previous studies investigating the whole brain [36], upper cervical cord area [37], and GM and WM measurements [23] using SIENAX [23, 36], volBrain, and FSL-Anat [23] and may imply that reliable atrophy measurements acquired from post-contrast images are possible across segmentation techniques.

Consistently, total GM, deep GM, and thalamic volume were between 3.06 and 17.39% higher in post-contrast images, and the same tendency was found for mean cortical thickness. Simultaneously, total WM volume was 1.74% lower in post-contrast images. The differences were systematic across all investigated measurements and exhibited no proportional bias. Inspecting cortical segmentations in more detail, we produced heatmaps highlighting within-subject cortical thickness differences in smaller cortical regions (Fig. 6). While smaller regions almost inevitably produce more variability than the larger regions that were the main focus of this work, these inspections showed that cortical thickness overestimation was a brain-wide phenomenon and that the overestimation in post-contrast images was not tied to large errors in any specific region but instead occurred throughout the brain.

These systematic differences in measured volumes and cortical thicknesses between pre- and post-contrast images mean that they should not be compared directly. Another study, using synthetic tissue mapping to measure brain tissue fractions [24], found a 1.1% increase in total WM fraction and an 0.7% decrease in GM fraction, in post-contrast images. Due to the methodological differences between that study and ours, it is difficult to assess the reason for the discrepancy in findings.

We could not identify any definite reason for the differences between pre- and post-contrast images. However, when visually inspecting images separately, some recurring soft failures in the FreeSurfer pipeline were found: First, the pial surface often expanded into extraparenchymal tissue, including components of dura or blood vessels as part of the cortex (Fig. 1b). These errors have been shown in areas where the dura or other structures like venous sinuses, lie tangentially in close proximity to the cortex or deep GM structures, leading to larger thickness and volume variability (Fig. 2) [38]. In the FreeSurfer processing stream, the failure to remove enough extraparenchymal tissue happens in the preliminary skull stripping step [39] and the accuracy of the pial surface can be improved by manually erasing the incorporated dura or blood vessels before rerunning analyses [40].

Another recurring soft failure concerned the pial surface. In the surface-based cortical reconstruction, the border between white and grey matter (the white surface) is delineated, following T1 intensity gradients. The pial surface is then grown from the white surface, which serves as a reference point [41]. In all images, but more frequently and severely in post-contrast images, the pial surface failed to follow the white surface, causing “looping” errors (Fig. 1a) and a subsequent incorrect enlargement of the cortical volume and thickness. To improve pial surface accuracy, it is recommended to check for any mistakes in the white surface, and possibly apply manual edits before rerunning analyses [40].

Although most cortical regions demonstrated an increase in cortical thickness in post-contrast images, there were a few exceptions, particularly in the medial part of the temporal lobe. In the entorhinal and parahippocampal gyrus, as well as in the temporal poles, the measured cortical thickness was in some patients thinner after GBCA administration. These regions have in common that they are relatively small and structurally complex, and on visual inspection of the errors, the constructed white surface did not correctly follow the intensity gradients, causing considerable errors in the white surface, and subsequently the pial surface, leaving out parts of the temporal pole (Fig. 3). Challenges in reconstructing parts of the temporal cortex are consistent with previous studies [31, 38, 40, 42], leading to increased variability of the local cortical thickness measurements [38].

The soft failures in the FreeSurfer pipeline occurred more often in post-contrast images in our data. This may be caused by the higher intensity in extraparenchymal structures in close proximity to the cortex or subcortical structures, causing disturbance and challenges in correctly separating different tissue types.

Skull stripping errors and other soft failures could in some selected regions be identified as the direct cause of increased cortical thickness or GM volume in post-contrast images. It is however uncertain if these errors can explain the systematic increase in almost all GM structures and the overall decrease in WM volume. Even in the absence of active lesions and GBCA leakage through disruptions in the BBB, GBCAs can still be expected to be present in the brain capillary network [24]. This presence may shorten the overall T1 relaxation time in all tissues, and possibly also affect intensity borders. In our MRIQC analyses, there was no difference between pre- and post-contrast images CNR, indicating that the separation of GM and WM tissue distributions was similar in pre- and post-contrast images. It should however be noted that extracting reliable noise estimates from parallel imaging is challenging.

Systematic effects dependent on the type of GBCA used, dosage, and delay time after administration are likely. In the data retrospectively used in the present study, these factors were not standardised, nor always stated, making them difficult to correct for. To further conclude on the reliability of post-contrast measurements, it is necessary for future research to investigate the possible systematic effects dependent on these variables.

This study is not without limitations. For a multicentre study, the number of patients included was limited, and patients were scanned on different scanners with varying sequence parameters and field strength. Furthermore, some details of the MRI protocol that may affect brain measurements (e.g., head coils [43, 44]) were in some cases neither stated nor retrospectively retrievable, making it difficult to evaluate the effect of these factors. Nonetheless, because the effect of field strength on atrophy measures has been studied before [45], we explored the results for 1.5 T and 3 T scanners separately. No systematic differences between the two field strengths regarding the different variabilities in the pre- and post-contrast images emerged, which could be due to small patient numbers and variable acquisition settings. Considering all these aspects, the fact that consistency between measurements before and after GBCA administration was observed across the different scanners, suggests that this behaviour is largely systematic. Future studies should investigate the effect of field strength and those of other aspects of image acquisition more systematically. Image analyses in this study were performed by FreeSurfer, while there are multiple other software packages that have been used in the MS literature. To focus the present work, we chose FreeSurfer because it allows both volumetric and cortical thickness measurements and has been widely used before in MS [46–50].

Finally, we did not perform any pre-processing to remove high-intensity regions (except for those in WM lesions, filled in the lesion filling process) from the post-gadolinium T1-weighted images. Future work should investigate whether removal or replacement of those regions, perhaps similar to the procedure followed as part of the lesion-filling process in the present work, could reduce the observed overestimation of grey matter.

## Conclusion

This study has demonstrated that reliable atrophy measurements may be obtained by FreeSurfer from post-contrast 3D T1-weighted images. A good to excellent consistency was observed between all investigated GM and WM measurements derived from images acquired before and after GBCA administration. However, due to the systematic overestimation of the GM in post-contrast images, measurements acquired from pre- and post-contrast images should not be compared directly, and measurements extracted from certain regions (e.g., the temporal pole) should be interpreted with caution. Furthermore, possible systematic effects dependent on GBCA dose and delay time after injections should be investigated.

## Supplementary Information

Below is the link to the electronic supplementary material.

Supplementary file1 (DOCX 4239 KB)

## Abbreviations

BBB: Blood–brain barrier
CNR: Contrast-to-noise ratio
CR: Contrast ratio
EDSS: Expanded Disability Status Scale
FFE: Fast field echo.
FLAIR: Fluid-attenuated inversion recovery
FLIRT: FMRIB´s Linear Image Registration Tool
FSL: FMRIB Software Library
GBCAs: Gadolinium-based contrast-agents
GM: Grey matter
ICC: Intra-class correlation coefficient
IQMs: Image quality metrics
LST: Lesion segmentation tool
mL: Millilitres
mm: Millimetre
MPRAGE: Magnetization-prepared rapid gradient-echo
MRI: Magnetic resonance imaging
MRIQC: MRI Quality Control Tool
ms: Millisecond
MS: Multiple sclerosis
RRMS: Relapsing–remitting MS
SD: Standard deviation
SNR: Signal-to-noise ratio
SPMS: Secondary progressive multiple sclerosis
SPSS: Statistical Product and Service Solutions
T: Tesla
T1: Inversion time
TE: Echo time
TR: Repetition time
WM: White matter

## Declarations

### Guarantor

The scientific guarantor of this publication is Hugo Vrenken, PhD.

### Statistics and biometry

No complex statistical methods were used in this paper.

### Study subjects or cohorts overlap

Some study subjects or cohorts have been previously reported in “ω-3 fatty acid treatment in multiple sclerosis (OFAMS study): *a randomised, double-blind, placebo-controlled trial “* by Torkildsen et al., Arch Neurol, 2012. 69(8): p. 1044–51, and in “Low vitamin D, but not tobacco use or high BMI, is associated with long-term disability progression in multiple sclerosis” by Wesnes et al., *Mult Scler Relat Disord*, 2021. 50: p. 102801.

### Methodology

• Retrospective

• cross-sectional

• multicentre study

## References

[1] Popescu V, Klaver R, Voorn P (2015). What drives MRI-measured cortical atrophy in multiple sclerosis?. Mult Scler.

[2] Horakova D, Dwyer MG, Havrdova E (2009). Gray matter atrophy and disability progression in patients with early relapsing-remitting multiple sclerosis: a 5-year longitudinal study. J Neurol Sci.

[3] Zivadinov R, Bergsland N, Dolezal O (2013). Evolution of cortical and thalamus atrophy and disability progression in early relapsing-remitting MS during 5 years. AJNR Am J Neuroradiol.

[4] Fisher E, Lee J-C, Nakamura K, Rudick RA (2008). Gray matter atrophy in multiple sclerosis: a longitudinal study. Ann Neurol.

[5] Sotirchos ES, Gonzalez-Caldito N, Dewey BE (2020). Effect of disease-modifying therapies on subcortical gray matter atrophy in multiple sclerosis. Mult Scler.

[6] Filippi M, Rocca MA, Pagani E (2014). Placebo-controlled trial of oral laquinimod in multiple sclerosis: MRI evidence of an effect on brain tissue damage. J Neurol Neurosurg Psychiatry.

[7] Gaetano L, Haring DA, Radue EW (2018). Fingolimod effect on gray matter, thalamus, and white matter in patients with multiple sclerosis. Neurology.

[8] Kuperberg GR, Broome MR, McGuire PK (2003). Regionally localized thinning of the cerebral cortex in schizophrenia. Arch Gen Psychiatry.

[9] Rosas HD, Liu AK, Hersch S (2002). Regional and progressive thinning of the cortical ribbon in Huntington’s disease. Neurology.

[10] Tustison NJ, Cook PA, Klein A (2014). Large-scale evaluation of ANTs and FreeSurfer cortical thickness measurements. Neuroimage.

[11] Schmidt MF, Storrs JM, Freeman KB (2018). A comparison of manual tracing and FreeSurfer for estimating hippocampal volume over the adult lifespan. Hum Brain Mapp.

[12] Cardinale F, Chinnici G, Bramerio M (2014). Validation of FreeSurfer-estimated brain cortical thickness: comparison with histologic measurements. Neuroinformatics.

[13] Eshaghi A, Prados F, Brownlee WJ (2018). Deep gray matter volume loss drives disability worsening in multiple sclerosis. Ann Neurol.

[14] Steenwijk MD, Geurts JJ, Daams M (2016). Cortical atrophy patterns in multiple sclerosis are non-random and clinically relevant. Brain.

[15] Calabrese M, Atzori M, Bernardi V (2007). Cortical atrophy is relevant in multiple sclerosis at clinical onset. J Neurol.

[16] Zijdenbos AP, Forghani R, Evans AC (2002). Automatic “pipeline” analysis of 3-D MRI data for clinical trials: application to multiple sclerosis. IEEE Trans Med Imaging.

[17] Nakamura K, Fox R, Fisher E (2011). CLADA: cortical longitudinal atrophy detection algorithm. Neuroimage.

[18] Smith SM, Zhang Y, Jenkinson M (2002). Accurate, robust, and automated longitudinal and cross-sectional brain change analysis. Neuroimage.

[19] Rovira À, Wattjes MP, Tintoré M (2015). MAGNIMS consensus guidelines on the use of MRI in multiple sclerosis—clinical implementation in the diagnostic process. Nat Rev Neurol.

[20] Saade C, Bou-Fakhredin R, Yousem DM, Asmar K, Naffaa L, El-Merhi F (2018). Gadolinium and multiple sclerosis: vessels, barriers of the brain, and glymphatics. AJNR Am J Neuroradiol.

[21] Rogosnitzky M, Branch S (2016). Gadolinium-based contrast agent toxicity: a review of known and proposed mechanisms. Biometals.

[22] Kanda T, Oba H, Toyoda K, Kitajima K, Furui S (2016). Brain gadolinium deposition after administration of gadolinium-based contrast agents. Jpn J Radiol.

[23] Hannoun S, Baalbaki M, Haddad R (2018). Gadolinium effect on thalamus and whole brain tissue segmentation. Neuroradiology.

[24] Warntjes JB, Tisell A, Landtblom AM, Lundberg P (2014). Effects of gadolinium contrast agent administration on automatic brain tissue classification of patients with multiple sclerosis. AJNR Am J Neuroradiol.

[25] Torkildsen O, Wergeland S, Bakke S (2012). omega-3 fatty acid treatment in multiple sclerosis (OFAMS Study): a randomised, double-blind, placebo-controlled trial. Arch Neurol.

[26] McDonald WI, Compston A, Edan G (2001). Recommended diagnostic criteria for multiple sclerosis: guidelines from the International Panel on the diagnosis of multiple sclerosis. Ann Neurol.

[27] Schmidt P, Gaser C, Arsic M (2012). An automated tool for detection of FLAIR-hyperintense white-matter lesions in Multiple Sclerosis. Neuroimage.

[28] Battaglini M, Jenkinson M, De Stefano N (2012). Evaluating and reducing the impact of white matter lesions on brain volume measurements. Hum Brain Mapp.

[29] Fischl B (2012). FreeSurfer. Neuroimage.

[30] Dale AM, Fischl B, Sereno MI (1999). Cortical surface-based analysis. I. Segmentation and surface reconstruction. Neuroimage.

[31] Desikan RS, Ségonne F, Fischl B (2006). An automated labeling system for subdividing the human cerebral cortex on MRI scans into gyral based regions of interest. Neuroimage.

[32] Esteban O, Birman D, Schaer M, Koyejo OO, Poldrack RA, Gorgolewski KJ (2017). MRIQC: advancing the automatic prediction of image quality in MRI from unseen sites. PLoS One.

[33] Zhang Y, Brady M, Smith S (2001). Segmentation of brain MR images through a hidden Markov random field model and the expectation-maximization algorithm. IEEE Trans Med Imaging.

[34] Giavarina D (2015). Understanding Bland Altman analysis. Biochem Med.

[35] Koo TK, Li MY (2016). A guideline of selecting and reporting intraclass correlation coefficients for reliability research. J Chiropr Med.

[36] Neacsu V, Jasperse B, Korteweg T (2008). Agreement between different input image types in brain atrophy measurement in multiple sclerosis using SIENAX and SIENA. J Magn Reson Imaging.

[37] Liu Y, Lukas C, Steenwijk MD (2016). Multicenter validation of mean upper cervical cord area measurements from head 3D T1-weighted MR imaging in patients with multiple sclerosis. AJNR Am J Neuroradiol.

[38] Han X, Jovicich J, Salat D (2006). Reliability of MRI-derived measurements of human cerebral cortical thickness: the effects of field strength, scanner upgrade and manufacturer. Neuroimage.

[39] Fennema-Notestine C, Ozyurt IB, Clark CP (2006). Quantitative evaluation of automated skull-stripping methods applied to contemporary and legacy images: effects of diagnosis, bias correction, and slice location. Hum Brain Mapp.

[40] McCarthy CS, Ramprashad A, Thompson C, Botti J-A, Coman IL, Kates WR (2015). A comparison of FreeSurfer-generated data with and without manual intervention. Front Neurosci.

[41] Fischl B, Dale AM (2000). Measuring the thickness of the human cerebral cortex from magnetic resonance images. Proc Natl Acad Sci USA.

[42] Oguz I, Cates J, Fletcher T et al (2008) Cortical correspondence using entropy-based particle systems and local features.10.1109/ISBI.2008.4541327

[43] Panman JL, To YY, van der Ende EL (2019). Bias introduced by multiple head coils in MRI research: an 8 channel and 32 channel coil comparison. Front Neurosci.

[44] Li H, Smith SM, Gruber S et al (2018) Combining multi-site/multi-study MRI data: linked-ICA denoising for removing scanner and site variability from multimodal MRI data. bioRxiv. 10.1101/337576:337576

[45] Battaglini M, Gentile G, Luchetti L (2019). Lifespan normative data on rates of brain volume changes. Neurobiol Aging.

[46] Guo C, Ferreira D, Fink K, Westman E, Granberg T (2019). Repeatability and reproducibility of FreeSurfer, FSL-SIENAX and SPM brain volumetric measurements and the effect of lesion filling in multiple sclerosis. Eur Radiol.

[47] Platten M, Martola J, Fink K, Ouellette R, Piehl F, Granberg T (2020). MRI-based manual versus automated corpus callosum volumetric measurements in multiple sclerosis. J Neuroimaging.

[48] Calabrese M, Rinaldi F, Grossi P, Gallo P (2011). Cortical pathology and cognitive impairment in multiple sclerosis. Expert Rev Neurother.

[49] Inglese M, Petracca M, Mormina E (2017). Cerebellar volume as imaging outcome in progressive multiple sclerosis. PLoS One.

[50] Chu R, Kim G, Tauhid S, Khalid F, Healy BC, Bakshi R (2018). Whole brain and deep gray matter atrophy detection over 5 years with 3T MRI in multiple sclerosis using a variety of automated segmentation pipelines. PLoS One.

