# Supplementary material for: The effect of gadolinium-based contrast-agents on automated brain atrophy measurements by FreeSurfer in patients with multiple sclerosis
Source: Eur Radiol. 2022 Jan 3;32(5):3576–87. doi: 10.1007/s00330-021-08405-8 (PMC9038813; doi:10.1007/s00330-021-08405-8)
Supplement: Supplementary file 1 — Supplementary file1 (DOCX 4239 KB) [file 330_2021_8405_MOESM1_ESM.docx]

| **Table e1.** MRI measurement values obtained by FreeSurfer v.6.0.1 | | | | | |
| --- | --- | --- | --- | --- | --- |
| MRI measurement | Mean value pre-contrast (SD) | Mean value post-contrast (SD) | Mean difference ^a^ (SD) | Percent increase/decrease (SD) | ICC (95% confidence interval) |
| Total grey matter volume (mL) | 602.49 (64.13) | 624.40 (65.60) | +21.91 (15.57)*** | +3.69 (2.64) % | 0.985 (0.965-0.994) |
| Total white matter volume (mL) | 454.28 (61.60) | 441.17 (59.54) | -13.11 (7.94)*** | -2.86 (1.64) % | 0.996 (0.990-0.998) |
| Total deep grey matter volume (mL) | 51.14 (5.86) | 55.36 (5.53) | +4.22 (2.68)*** | +8.58 (6.14) % | 0.942 (0.859-.976) |
| Left Thalamus volume (mL) | 6.44 (0.87) | 7.89 (1.02) | +1.44 (0.62)*** | +22.90 (11.06) % | 0.880 (0.711-.950) |
| Right Thalamus volume (mL) | 6.26 (0.89) | 7.33 (0.99) | +1.07 (0.58)*** | +17.67 (10.07) % | 0.896 (0.749-.957) |
| Mean cortical thickness left hemisphere (mm) | 2.35 (0.17) | 2.58 (0.18) | +0.23 (0.04)*** | +9.60 (1.91) % | 0.984 (0.962-0.993) |
| Mean cortical thickness right hemisphere (mm) | 2.35 (0.17) | 2.60 (0.17) | +0.25 (0.06)*** | +10.74 (2.73) % | 0.971 (0.930-0.988) |

**Supplementary material**

Abbreviations: SD = standard deviation, ICC = intra-class correlation coefficient for consistency, mL = millilitre, mm = millimetre

^a^ Paired t-test

***p<0.0001

| **Table e2.** MRI measurement values obtained from 1.5 and 3.0 T scanners. | | | | | | |
| --- | --- | --- | --- | --- | --- | --- |
| Field strength | 1.5 T (17 patients) | | | 3.0 T (5 patients) | | |
| MRI measure | Mean value pre-contrast (SD) | Mean value post-contrast (SD) | Mean difference^a,b^ (SD) | Mean value pre-contrast (SD) | Mean value post-contrast (SD) | Mean difference^a,b^ (SD) |
| Total grey matter volume (mL) | 593.44 (63.46) | 616.75 (63.76) | 23.32 (13.73)** | 633.46 (52.95) | 632.48 (48.78) | -0.98 (7.35) |
| Total white matter volume (mL) | 459.22 (65.22) | 451.68 (62.34) | -7.55 (6.88)** | 449.71 (59.39) | 438.60 (52.16) | -11.11 (9.04) |
| Total deep grey matter volume (mL) | 51.11 (6.16) | 54.05 (6.04) | 2.93 (1.50)** | 53.14 (5.20) | 57.07 (3.26) | 3.93 (3.45) |
| Left Thalamus volume (mL) | 6.48 (0.94) | 7.49 (1.18) | 1.02 (0.49)** | 6.47 (1.00) | 7.87 (0.72) | 1.41 (0.34)* |
| Right Thalamus volume (mL) | 6.27 (1.01) | 6.90 (.88) | 0.64 (0.45)** | 6.72 (0.92) | 7.84 (0.70) | 1.12 (0.53)* |
| Mean cortical thickness left hemisphere (mm) | 2.28 (0.15) | 2.45 (0.15) | 0.18 (0.06)** | 2.46 (0.04) | 2.60 (0.05) | 0.14 (0.04)* |
| Mean cortical thickness right hemisphere (mm) | 2.28 (0.14) | 2.46 (0.14) | 0.17 (0.06)** | 2.47 (0.04) | 2.60 (0.04) | 0.14 (0.03)* |

Abbreviations: MRI = magnetic resonance imaging, T = tesla, SD = standard deviation, mL = millilitres, mm = millimetre.

^a^ Paired t-test

^b^The results of the performed t-tests for each field strength are viewed as exploratory and should not be directly compared, as patients were only scanned on one scanner. Furthermore, at total of 5 different 1.5T scanners, and 2 different 3T scanners were used.

***p*<0.001, **p*<0.01

**Figure e1.** *Bland-Altman plots, illustrating a systematic difference in global (A) and regional (B) MRI measurements, but no proportional bias.*


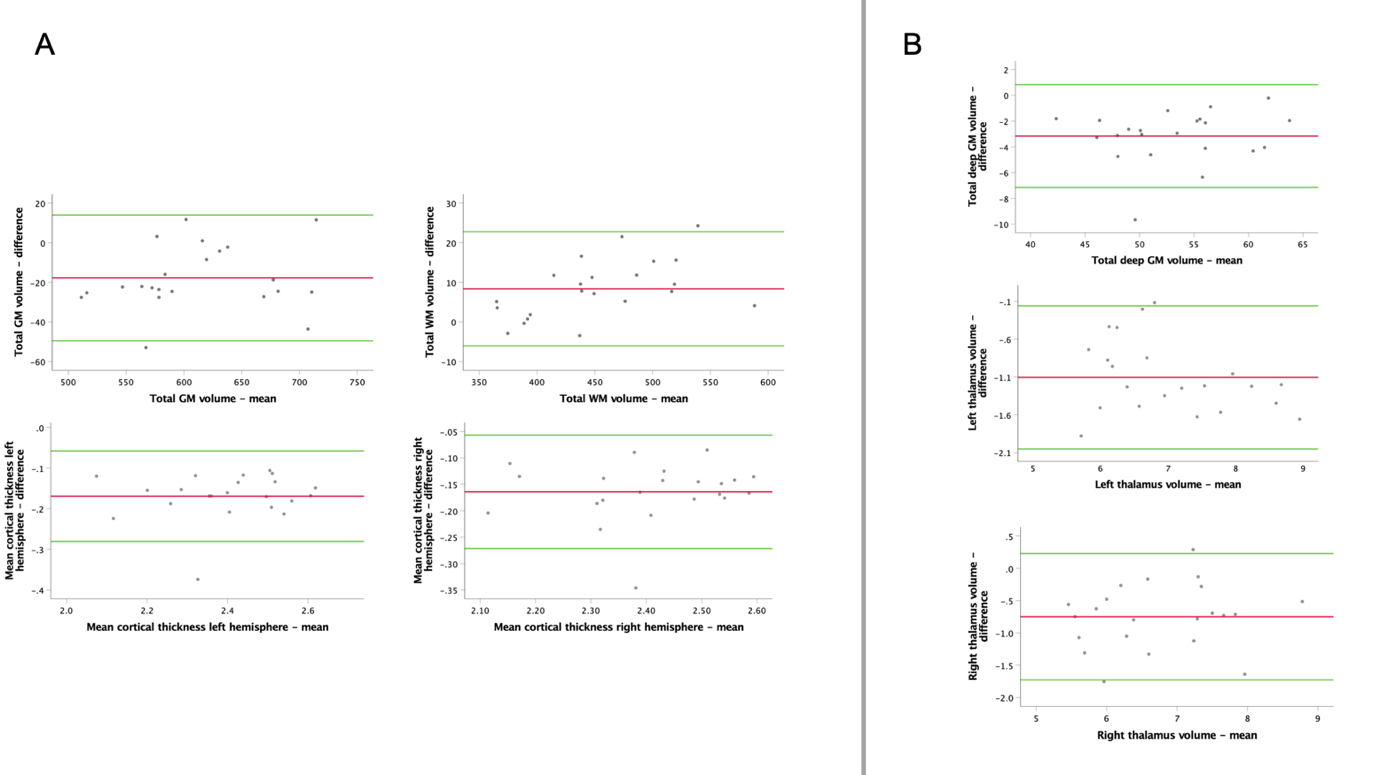


**eAppendix 1**

**MRI protocol**

Magnetic field strength: Preferably 3 Tesla (T) if available, alternatively 1,5 T

Standard head coil

Positioning: supine

**Protocol for MR cerebrum**

(Siemens Prisma 3T indicated in brackets; for other configurations, see ADNI/Helse Vest protocols in links below)

1. Localizer
   (Prisma: 3 slices, gap 20%, sagittal (SAG), field of view (FOV) 300mm, matrix (M)=256x256, echo time (TE)/ repetition time (TR)=4/8.6 ms, flip angle (FA) 20, Avg 2)
2. Alternatively quick T2 transverse (TRA)/SAG recording to localize corpus callosum (parameters of choice)
3. Echo planar (EP) diffusion-weighted imaging (DWI), TRA, 5mm slice/ 20 % gap, resolution: (e.g., 1.5T 1.6x1.6x5 mm3)

Angled after the anterior and posterior part of corpus callosum/the hard palate. b= 0, 1000. Apparent diffusion coefficient (ADC) map.

(Prisma: Resolve, TRA, 28sl, gap 30 %, FOV 220mm, TE/TR=54/3700ms, M=160x160, 4mm).

1. T2 3D fluid-attenuated inversion recovery (FLAIR) SAG resolution: 1x1x1mm3. Reconstructed in three planes with 0% gap. Alternative to 3D is 2D recording: 2D coronal (COR) FLAIR and SAG T2 FLAIR

(Prisma: Space-IR, SAG, 192 sl, FOV = 256mm, TE/TR = 386/5000ms, M=256x256)

1. T1 3D magnetization prepared rapid gradient echo (MPRAGE) SAG resolution: 1x1x1mm3. Reconstructed in three planes with 0 % gap. Recordings are done 5 minutes after intravenous contrast injection. (I possible also the same T1 recorded before intravenous contrast injection for optimal segmentation) (Prisma: MPRAGE, SAG, TE/TR/TI= 2.28/1800/900 ms, M=256x256, FA=8)
2. If available 3T MRI; diffusion tensor imaging (DTI) recording: standard echo-planar-imaging DTI (b=0,1000), 6-64 directions, e.g., 5 b=0 and 25 directions b=1000.

Links for protocols:

Helse Vest RHF (the Western Norway Regional Health Authority): <https://helse-vest.no/seksjon/radiologiske-prosedyrar/Documents/MR%20nevro/MR%20ms.pdf>

ADNI (Alzheimer’s Disease Neuroimaging Initiative): <http://adni.loni.usc.edu/methods/documents/mri-protocols/>

**eAppendix 2**

**Summary of FreeSurfer procedures**

FreeSurfer uses a combined volume-based and surface-based approach to automatically segment the brain and to calculate volume and average cortical thickness in defined regions of interest. Included in the preprocessing steps are removal of non-brain tissue, registration of the structural volume with the Talairach atlas, assigning neuroanatomical labels to cortical and subcortical regions [1], intensity normalization, tessellation of the white/grey matter boundary, automated topology correction and surface deformation routines to optimally create white/grey and grey/cerebrospinal fluid surface models. These surface models are inflated and then registered to a spherical atlas, to match individual cortical folding patterns to cortical geometry across subjects, based on gyral and sulcal structure [2]. The closest distance from the white/grey boundary to the grey/cerebrospinal fluid boundary at each surface’s vertex is defined as the thickness. Lastly, the final cortical and subcortical GM volumes are automatically assigned neuroanatomical labels based on probabilistic global and local spatial information estimated from a manually labelled training set [1,3].

**References**

1 Fischl B, Salat DH, Busa E et al (2002) Whole brain segmentation: automated labeling of neuroanatomical structures in the human brain. Neuron 33:341-355

2 Desikan RS, Ségonne F, Fischl B et al (2006) An automated labeling system for subdividing the human cerebral cortex on MRI scans into gyral based regions of interest. Neuroimage 31:968-980

3 Fischl B, van der Kouwe A, Destrieux C et al (2004) Automatically parcellating the human cerebral cortex. Cereb Cortex 14:11-22
